# Supplementary material for: Candidatus Dermatophostum as a novel genus of polyphosphate-accumulating organisms for high-strength wastewater treatment
Source: ISME J. 2026 Feb 21;20(1):wrag032. doi: 10.1093/ismejo/wrag032 (PMC12978653; doi:10.1093/ismejo/wrag032)
Supplement: supplementary_files_wrag032 [file supplementary_files_wrag032.zip › Novel_PAO_SI_20260206_-_plain_wrag032.docx]

**SUPPLEMENTARY MATERIAL for**

***Candidatus* Dermatophostum as a Novel Genus of Polyphosphate-Accumulating Organisms for High-Strength Wastewater Treatment**

***Corresponding author contact:**

Dr. Feng Ju

Associate Professor (Principal Investigator)

Environmental Microbiome and Biotechnology Laboratory (EMBLab)

Westlake University

Tel.: 571-87963205 (lab), 571-87380995 (office)

E-mail: [jufeng@westlake.edu.cn](mailto:jufeng@westlake.edu.cn)

**Figure Legends**

Figure S1 The performance of EBPR reactor enriched with *Dermatophilaceae* PAOs on day 180.

Fig. S2 Temporal profiles of total nitrogen, ammonia and total organic carbon removal in the EBPR reactor during 266 days of operation.

Fig. S3 Heatmap of average nucleotide identities (ANI) among *Dermatophilaceae* MAGs and reference genomes.

Fig. S4 Heatmap of average nucleotide identities (ANI) among clade 1, 2, 3 and 6 *Dermatophilaceae* PAOs.

Fig. S5 Heatmap of average nucleotide identities (ANI) among clade 4 and 5 *Dermatophilaceae* PAOs.

Fig. S6 Predicted local distance difference test (pLDDT) confidence scores of pit transport proteins in *Ca.* Dermatophostum ammonifactor.

Fig. S7 Blastp analysis of the predicted NrfA protein.

Fig. S8 Gene expression and dynamic patterns of core metabolism in *Dermatophilaceae* PAOs in lab-scale EBPR system.

Fig. S9 Spearman correlation of PAO genus belonging to *Dermatophilaceae* family: Tetrasphaera_A, *Ca*. Lutibacillus, *Ca*. Phosphoribacter, *Ca*. Dermatophostum, and *Phycicoccus_A*.

**Supplementary Method**

Method S1 Reactor operation and routine monitoring

Method S2 FISH experiment

Method S3 DNA extraction, library construction, metagenomic sequencing

Method S4 Metagenome pretreatment, assembly, and binning

Method S5 Metagenome-assembled genome analysis and functional annotation

Method S6 Text S6 RNA isolation, metatranscriptomic sequencing, and bioinformatics analysis

Method S7 Protein Structure Prediction, Ligand Docking, and Conservation Analysis

**Table Legends**

Table S1 FISH probe used for detection of different PAO groups in this study.

**Supplementary Note**

Note S1 Organic substrate transport *Dermatophilaceae* PAOs


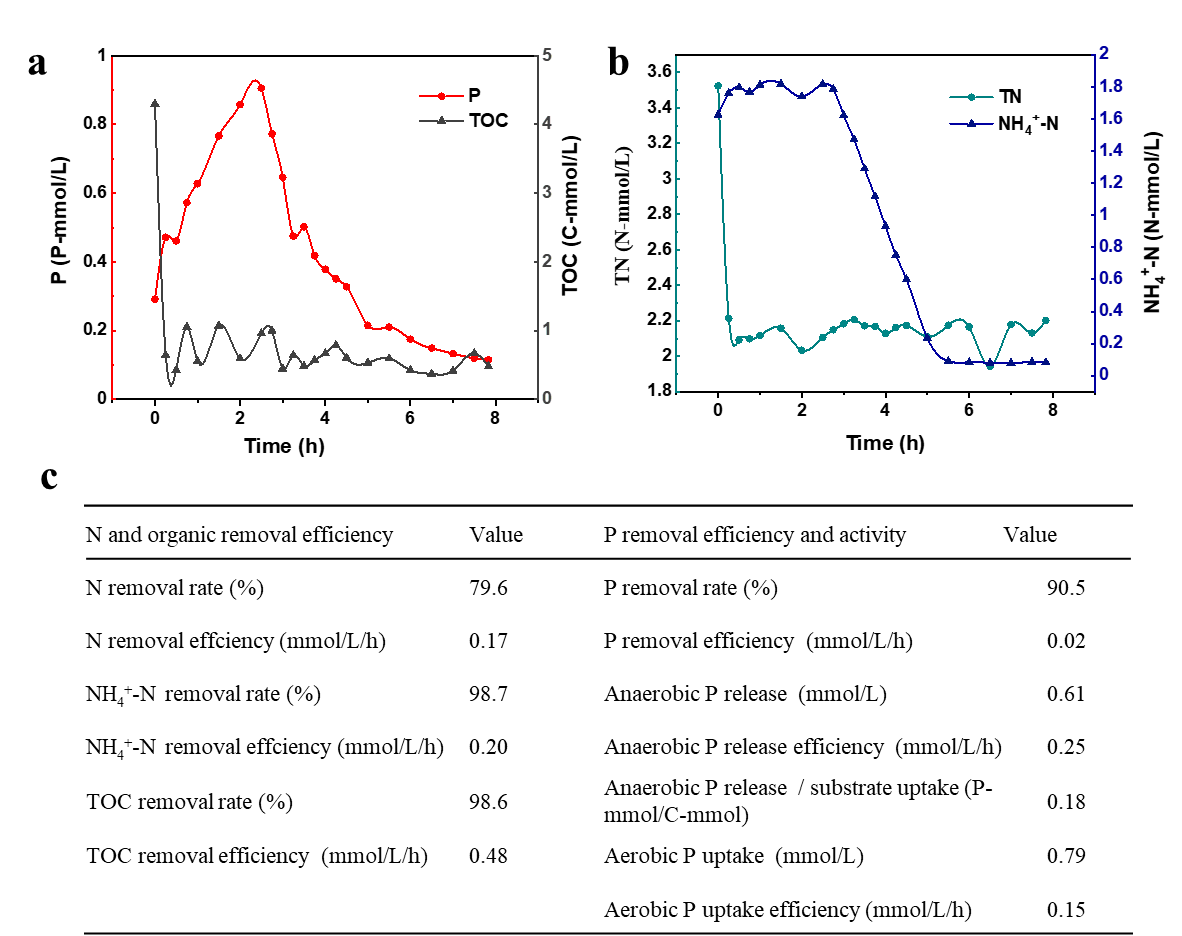


Fig. S1 The performance of EBPR reactor enriched with *Dermatophilaceae* PAOs on day 180. (a) the phosphorus (P) and total organic carbon (TOC) concentration during an EBPR cycle. (b) the total nitrogen (TN) and ammonia (NH₄⁺-N) concentration during an EBPR cycle. (c) the nutrient (nitrogen and phosphorus) and organic removal efficiency and activity of EBPR microbiome. The 8-h cycle of the EBPR reactor operation consisted of 150-min anaerobic treatment, 320-min aerobic treatment, 5-min settling, and 5-min water discharge & feeding.


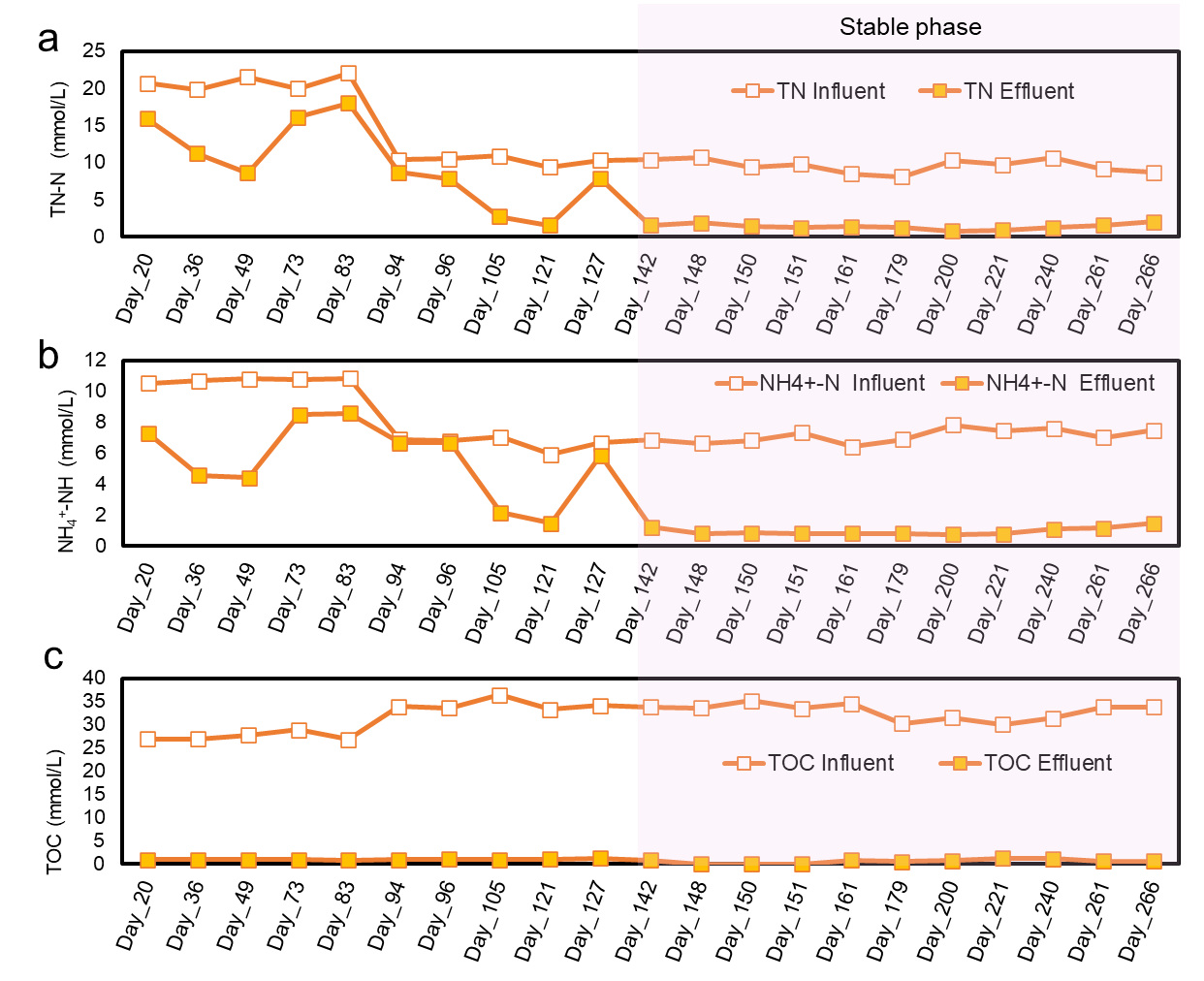


Fig. S2 Temporal profiles of total nitrogen, ammonia and total organic carbon removal in the EBPR reactor during 266 days of operation. (a) Total nitrogen (TN), (b) ammonium (NH₄⁺-N), and (c) total organic carbon (TOC) concentrations in the influent and effluent.


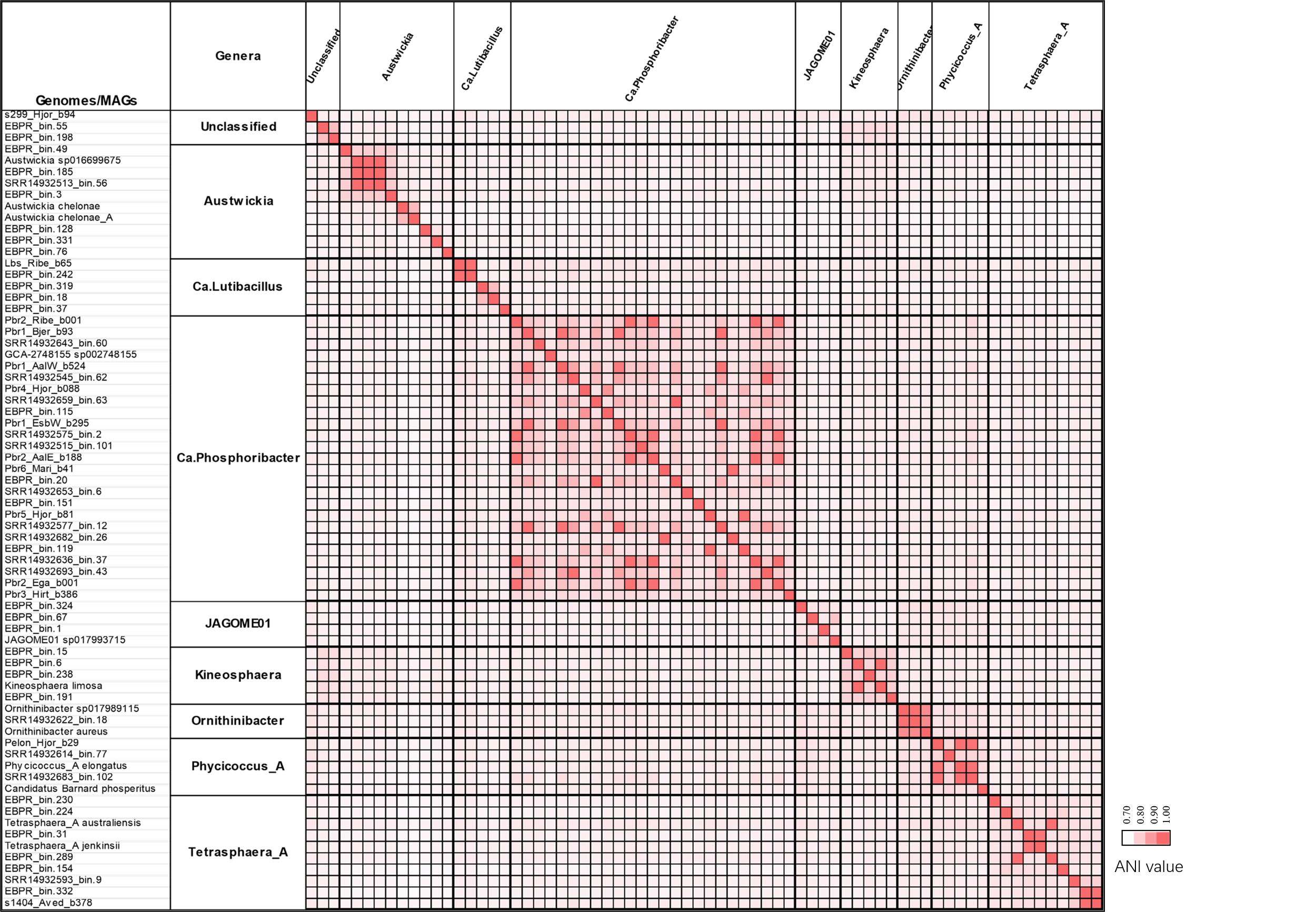


Fig. S3 Heatmap of average nucleotide identities (ANI) among Dermatophilaceae MAGs and reference genomes. Pairwise ANI values were calculated for 46 MAGs reconstructed in this study. Taxonomic assignments were performed using GTDB-Tk. MAGs are grouped by genus, and ANI values are visualized with a red gradient scale, where darker colors indicate higher nucleotide similarity. The results support genus-level delineation within Dermatophilaceae and highlight the JAGOME01 (*Ca.* Dermatophostum) as a distinct, previously uncharacterized genus.


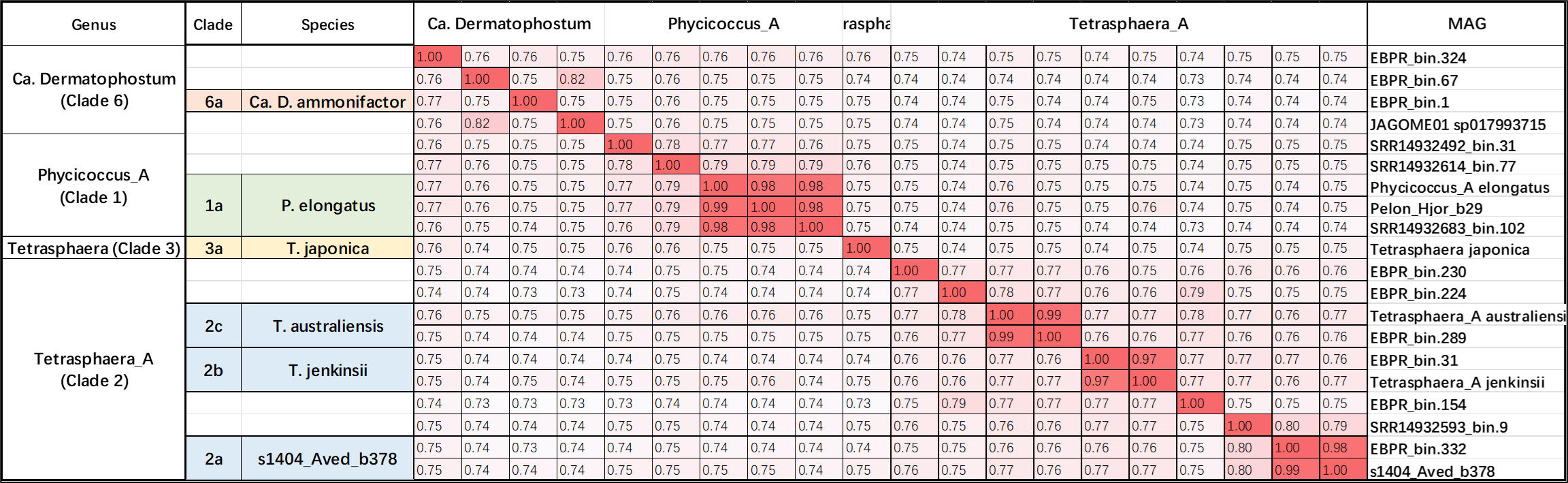


Fig. S4 Heatmap of average nucleotide identities (ANI) among clade 1, 2, 3 and 6 *Dermatophilaceae* PAOs. Taxonomic assignments were performed using GTDB-Tk. MAGs are grouped by genus, and pairwise ANI values are visualized with a red gradient scale, where darker colors indicate higher nucleotide similarity. Species-level classifications are labeled next to each clade. The results reveal clear intra-clade subdivisions, consistent with species-level genomic delineations.


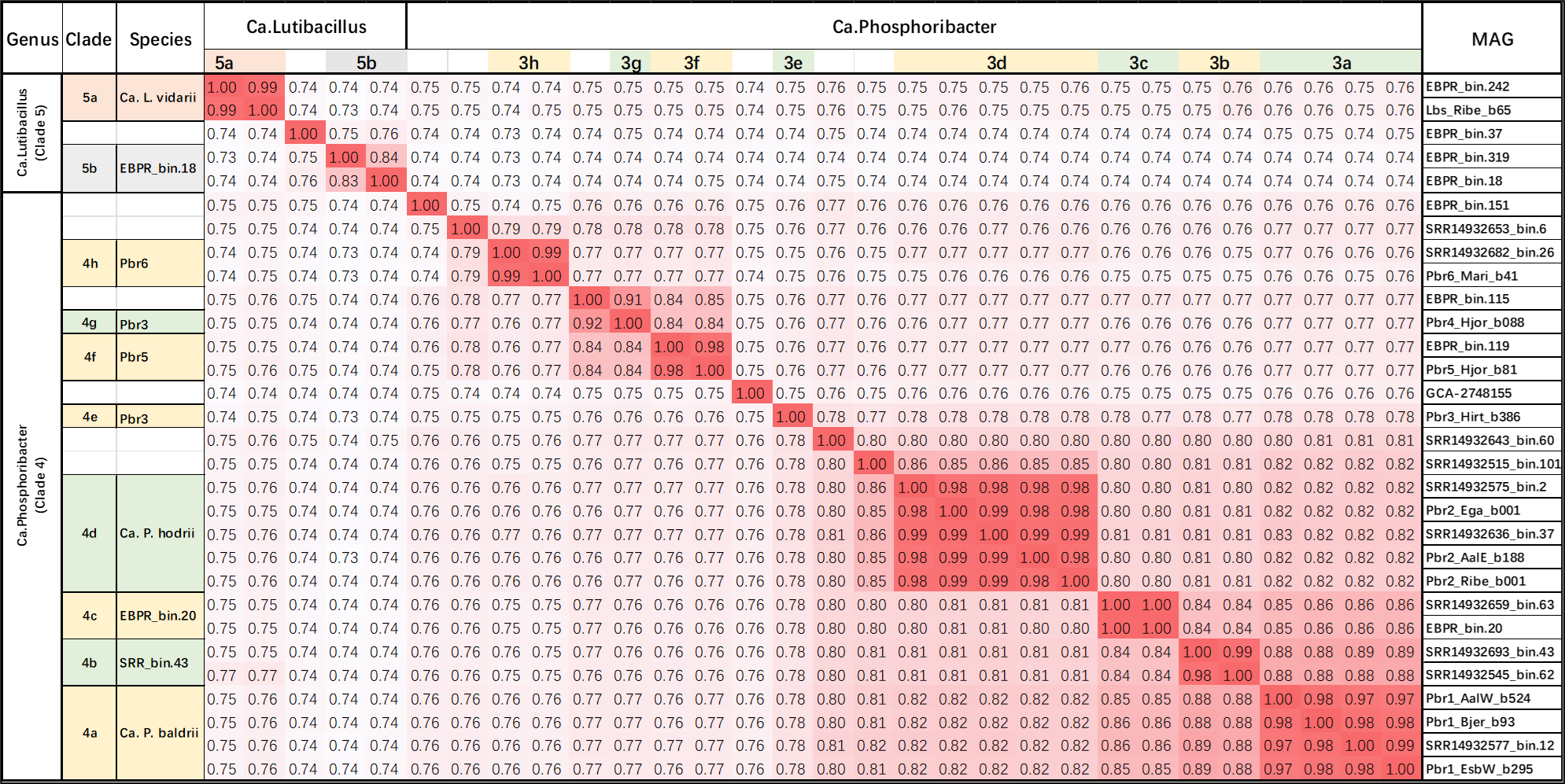


Fig. S5 Heatmap of average nucleotide identities (ANI) among clade 4 and 5 *Dermatophilaceae* PAOs. Taxonomic assignments were performed using GTDB-Tk. MAGs are grouped by genus, and pairwise ANI values are visualized with a red gradient scale, where darker colors indicate higher nucleotide similarity. Species-level classifications are labeled next to each clade. The results reveal clear intra-clade subdivisions, consistent with species-level genomic delineations.


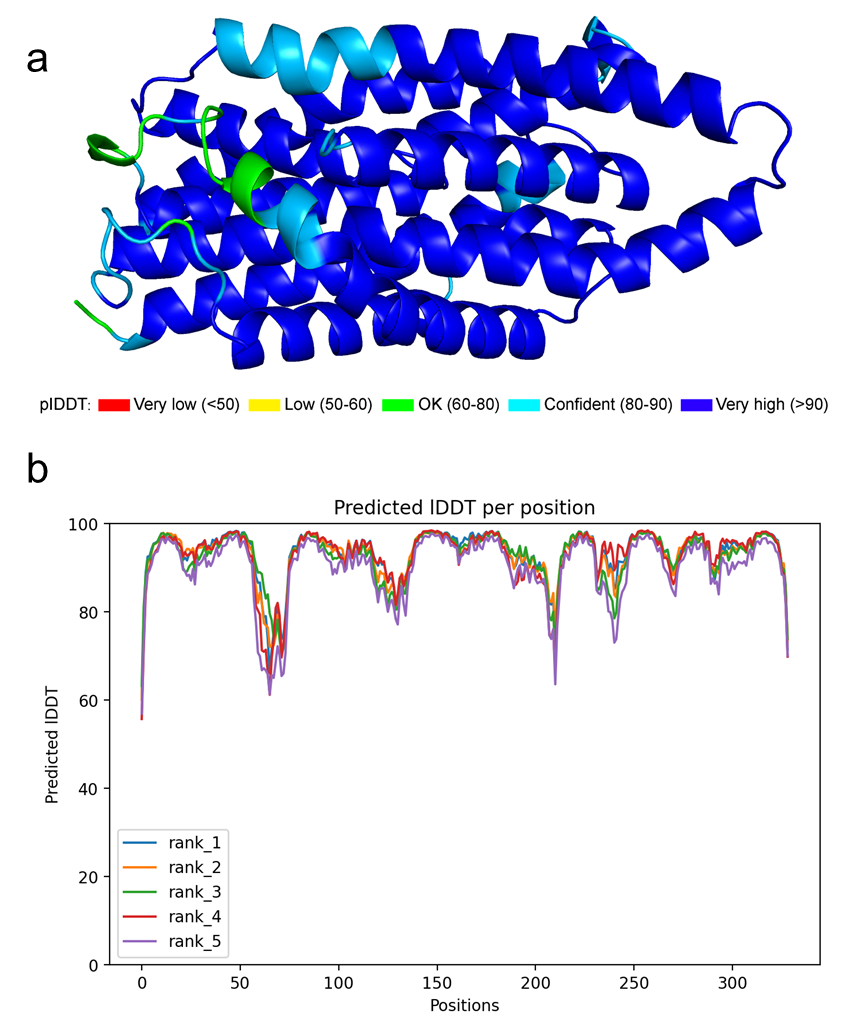


Fig. S6 Predicted local distance difference test (pLDDT) confidence scores of *pit* transport proteins in *Ca.* Dermatophostum ammonifactor. (a) The predicted structure was shown as a ribbon diagram, with the pLDDT score for each residue represented by a color scale. Red indicates very low confidence (<50), yellow represents low confidence (50-60), green indicates OK confidence (60-80), cyan shows confident regions (80-90), and blue highlights very high confidence (>90). (b) The predicted IDDT (internal distance difference test) score across all positions in the protein, with different ranks (1 to 5) corresponding to different predictions.


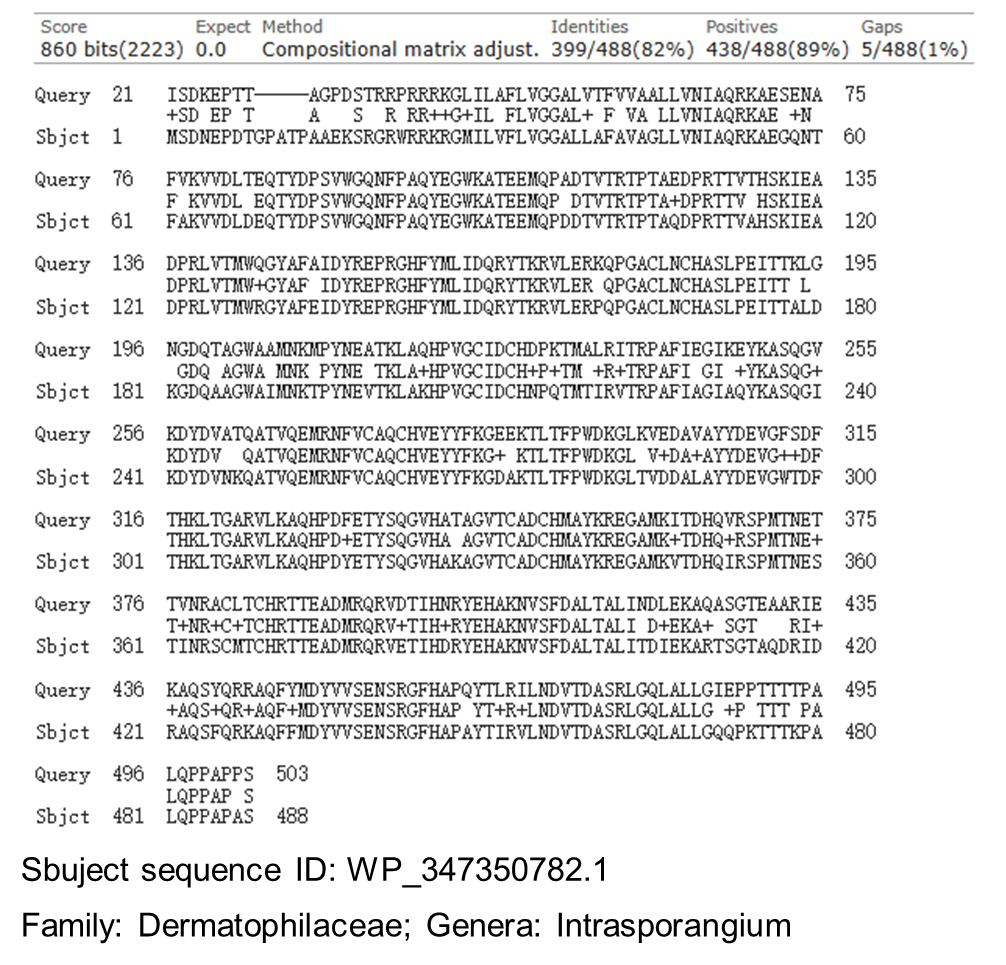


Fig. S7 Blastp analysis of the predicted NrfA protein. Blastp analysis was performed to compare the predicted NrfA protein encoded by *Ca.* D. ammonifactor with functionally validated homologs in the NCBI database. The predicted sequence showed high similarity to a validated homolog from the same family, with 96% coverage and 81.8% amino acid identity. Sequence alignment confirmed strong conservation of the catalytic motifs, supporting the functional annotation of NrfA in Ca. D. ammonifactor.

Fig. S8 Gene expression and dynamic patterns of core metabolism in *Dermatophilaceae* PAOs in lab-scale EBPR system. (a) Expression profiles of key genes involved in phosphorus metabolism. (b) Expression of genes related to nitrogen metabolism. (c) Transcription of genes associated with organic substrate transport (amino acids and sugars), fermentation, and storage polymer synthesis (polyhydroxyalkanoates and glycogen). These data include four representative MAGs, namely *T.* EBPR_bin.31 (clade 2), *Ca.* P. EBPR_bin.20 (clade 4), Ca. L. EBPR_bin.18 (clade 5), and Ca. D. ammonifactor (clade 6). Each column corresponds to a sampling stage during reactor operation: S1 (day 9-23), S2 (day 54-66), S3 (day 113-127); S4 (day 170-179); S5 (day 224-240). Each row represents the gene expression of a genome. Color intensity represents the log-transformed normalized expression level, measured in fragments per kilobase of transcript per million mapped reads (FPKM). White (empty) boxes indicate the absence of the corresponding gene in the genome. Pathway schematics illustrate the functional context of key genes. Detailed expression values are provided in Supplementary Dataset S7.


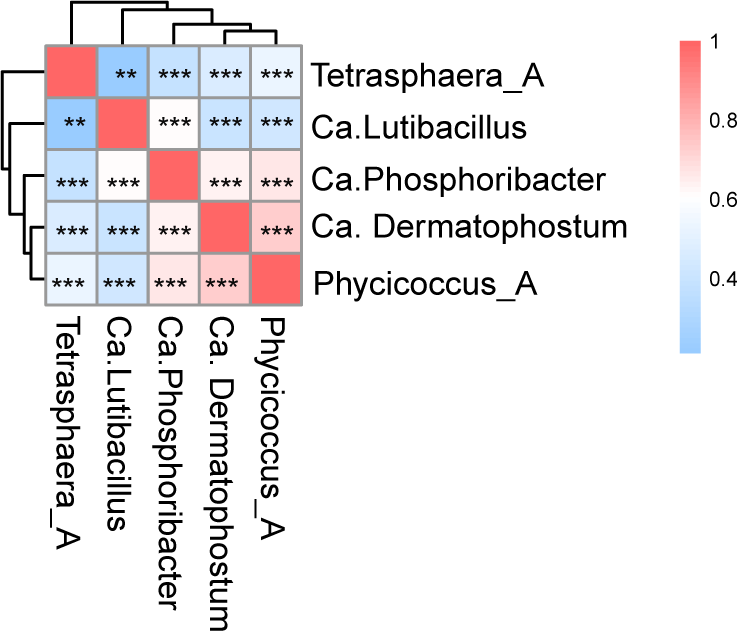


Fig. S9 Spearman correlation of PAO genus belonging to *Dermatophilaceae* family: *Tetrasphaera_A*, *Ca*. Lutibacillus, *Ca*. Phosphoribacter, *Ca*. Dermatophostum, and *Phycicoccus_A*. The T test was used to determine whether indices between the two groups were significant.

**Supplementary Methods**

**Method S1 Reactor operation and routine monitoring**

The operational cycle of the SBR consisted of an anaerobic phase (150 min), an aerobic phase (320 min), a settling phase (5 min), and a decant phase (5 min). The reactor was fed a mixture of amicase, glucose and sodium acetate as carbon sources, and a mixture of K_2_HPO_4_ and KH_2_PO_4_ as P sources. The reactor was inoculated with activated sludge from a WWTP in Hangzhou, China. The SBR was operated for more than 260 days, during which 1-5 mg/L allylthiourea (C_4_H_8_N_2_S, ATU) was added between 74-200 days to facilitate the enrichment of PAOs, as priorly described [1, 2]. During reactor operation, influent and effluent samples were collected according to practical operational needs (1-2 times per week) for the analysis of nutrient concentrations (i.e., PO_4_^3-^-P, NH_4_^+^-N and TN) and organic carbon (i.e., TOC), as described previously [2].

**Method S2 FISH experiment**

The biomass collected from the reactor were fixed by adding 37% formaldehyde solution (the ratio between biomass and formaldehyde is 10:1) then held at 4 ^o^C for 2 hours. After centrifugation followed by a twice wash (suspending samples in the mix solution of phosphate buffered saline (PBS) and ethanol (volume 1:1)), the resuspended biomass was collected then loaded into the glass slides for incubation. The incubation condition was 65 ^o^C, 1 hour, then the slides were immersed in 50% ethanol, 80% ethanol and 100% ethanol in turn for dehydration. After dried in weak air flow, the probes were loaded with prepared hybridization buffer (35% formamide) for hybridization. The hybridization condition was 46 ^o^C for 12 hours. The EUBmix probe that composed three probes (EUB338-1, EUB338-2 and EUB338-3) was used to target the entire bacterial community. The TETmix probe that composed Tet1-266, Tet2-892, Tet2-174 and Tet3-654 probe was used to target the 16S rRNA gene of *Dermatophilaceae* PAOs. The detailed information of FISH probe can be found in Table S1. After washing using prepared wash buffer followed by air dry, the probe labeled biomass was examined using a laser scanning confocal microscope (Zeiss, Zeiss LSM800, Germany).

**Method S3 DNA extraction, library construction, metagenomic sequencing**

For each sample, 1.5 mL of sludge was centrifuged, and the resulting pellet was subjected to DNA extraction using the FastDNA spin kit for soil (MP Biomedicals, USA), following the manufacturer’s instructions. DNA quality was assessed via agarose gel electrophoresis, and DNA concentration was quantified using a NanoDrop 2000 (ThermoFisher Scientific, Waltham, MA, USA). Metagenomic DNA library were prepared using the NEB Next® Ultra™ DNA Library Prep Kit for Illumina (NEB, USA). Sequencing was performed on the Illumina Novaseq platform using a 150 bp paired-end sequencing strategy at Novogene (Beijing, China). A total of 291.63 Gbp of metagenomic sequencing data was generated from the time series reactor samples (n = 16) and the detailed information is available in Supplementary Dataset S1.

**Method S4 Metagenome pretreatment, assembly, and binning**

The raw metagenomic data generated from the laboratory EBPR system underwent quality control using FastQC [3] v0.11.7 and MultiQC [4] v1.7. Raw reads were filtered to remove sequencing adapters and low-quality reads using Fastp [5] v0.19.7 and PRINSEQ-lite [6] v0.20.4. Two assembly strategies, including single sample assembly and multiple samples co-assembly, were employed in this study. For single sample assembly, quality-controlled reads from each sample were assembled separately using SPAdes [7] v3.9.0 with the parameters: -meta -k 21,33,55. Then, contigs from each sample were then binned independently to generate metagenome-assembled genomes (MAGs) using three binning software (i.e., MetaBAT2 [8], MaxBin [9], and CONCOCT [10]) in the MetaWRAP pipeline [11] v1.3.0 with default parameters. To recover the genome of *Dermatophilaceae* PAOs from global WWTPs, a metagenomic dataset (2.72 Tbp) was downloaded from the NCBI SRA database (PRJNA509305). This dataset includes activated sludge metagenomes from 226 WWTPs across 16 countries on 6 continents. The single sample assembly and binning strategy described above was applied to recover MAGs from this global dataset. For multiple samples co-assembly, metagenomic reads from all EBPR samples (n=16) were co-assembled using MEGAHIT [12] with default parameters. The resulting contigs were imported into MetaBAT2 [8] to recover MAGs with default settings.

**Method S5 Metagenome-assembled genome analysis and functional annotation**

The recovered MAGs from different assembly strategies were dereplicated using dRep [13] v2.3.2 with the thresholds of 90% Mash similarity for the primary clustering and 95% ANI for the secondary clustering, ≥70% completeness, and ≤10% contamination to remove the replicated MAGs. The relative abundance of the dereplicated MAGs was calculated using CoverM with the “relative abundance” method in the “genome” mode (v0.2.0, https://github.com/wwood/CoverM), which calculates abundance based on read coverage across all dereplicated MAGs. Genome taxonomy was determined using GTDB-Tk [14] v2.1.0 and its dependencies Prodigal [15] v2.6.3, HMMER [16] v3.1b2, pplacer [17] v1.1, FastANI [18] v1.32, FastTree [19] v2.1.9 and Mash [20] v2.2. Genome quality was assessed using CheckM [21] v1.2.0 with the ‘lineage_wf’ parameter.

MAGs assigned to the *Dermatophilaceae* family were selected for further comparative genomics analysis and functional annotation. The average nucleotide identity (ANI) between pairwise MAGs assigned to *Dermatophilaceae* was calculated using pyani [22] v0.2.12. The resulting ANI matrix was processed and visualized using R [23] v4.1.0 with the pheatmap and RColorBrewer libraries. Meanwhile, protein-coding genes were predicted from the contigs of the MAGs using Prodigal [15] v2.6.3 and functionally annotated using Prokka [24] v 1.14.6. To enhance the accuracy and completeness of functional annotation, additional annotation was performed using KofamScan [25] based on Kyoto Encyclopedia of Genes and Genomes (KEGG) database [26] using stringent settings (enforcing KO-specific thresholds and an E-value cutoff of <1e-10). Functional annotation of the MAG proteins was also performed with EnrichM v0.5.0 (<https://github.com/geronimp/enrichM>), which uses Diamond [27] v0.9.22.123 to blast the MAG protein sequences against a KO-annotated uniref100 database. For this step, strict filtering criteria were applied, requiring an E-value <1e-10, sequence identity >50%, and alignment coverage >80%. The above annotated results were manually cross-validated (union of annotations) and used for reconstructing metabolic pathways. Metabolic pathways in MAGs were reconstructed by referring to KEGG pathway using KEGG Mapper [28] v4.1 and visualized in BioRender (<https://biorender.com/>).

To assess the functional annotation of the predicted NrfA protein from *Ca.* D. ammonifactor, BLASTp searches were performed against both the NCBI non-redundant protein sequence database and the UniProt database, with an e-value cutoff of 1e-10. The predicted NrfA sequence exhibited 96% coverage and 81.8% amino acid identity to a homolog in NCBI, and 78.5% identity with 87.9% positives to a homolog from Intrasporangium calvum in UniProt (481 amino acids, Score 2048 bits, E-value 0), supporting the reliability of its annotation.

**Method S6 RNA isolation, metatranscriptomic sequencing, and bioinformatics analysis**

RNA quality was determined by 1.5% agarose gel electrophoresis, RNA concentration was measured using NanoDrop 2000 (ThermoFisher Scientific, Waltham, MA, USA), and RNA integrity was evaluated using an Agilent 2100 Bioanalyzer (Agilent, USA). After removal of ribosomal RNA using TIANSeq rRNA Depletion Kit (NR201-T5) (TIANGEN, China), the rRNA-depleted RNA was used for reverse transcription and cDNA library construction using the TruSeq Stranded mRNA Kit (Illuminia, USA), following the manufacturer’s instructions. The constructed cDNA libraries were sequenced on the Illumina’s NovaSeq platform using a paired-end (2 × 150) sequencing strategy at the Personal Biotechnology Co., Ltd. (Shanghai, China).

Raw metatranscriptomic reads were processed quality control prior to further analysis. Sequencing adapters were trimmed using cutadapt [29] (v1.17), and low-quality reads were removed using a sliding-window algorithm in fastp [5] (v0.20.0). Ribosomal RNA reads were removed with sortMeRNA [30] using the six default rRNA reference database. The remaining high-quality reads were mapped to the MAG using hisat2 [31] with default parameters. Read counts were calculated using HTseq [32] with the ‘intersection-strict’ parameter and normalized to transcripts per kilobase million (TPM) using stringtie [33].

**Method S7 Protein Structure Prediction, Ligand Docking, and Conservation Analysis**

Protein structure prediction was performed using AlphaFold2, implemented via the ColabFold pipeline [34], which integrates MMseqs2-based multiple sequence alignment and optimized inference settings. The quality of predicted models was assessed based on per-residue pLDDT scores, with structures exhibiting average scores above 90 considered highly reliable for downstream analysis. Predicted protein structures were subjected to ligand-binding site prediction and molecular docking using CB-Dock2 [35], a cavity detection-based docking server. The tool automatically identifies potential binding pockets and performs blind docking using default parameters. Binding pockets were evaluated based on cavity volume and predicted binding affinity. To assess the evolutionary conservation of amino acid residues, ConSurf [36] analysis was conducted. Homologous sequences were identified using HMMER against the UniRef90 database, and multiple sequence alignment was performed with MAFFT. Conservation scores were calculated using the Bayesian method and mapped onto the predicted 3D structures to highlight functionally important or evolutionarily conserved regions.

**Table S1.** FISH probe used for detection of different PAO groups in this study.

| Primer | Sequence (5’ to 3’) | Target group | Label | Ref. |
| --- | --- | --- | --- | --- |
| EUB338-1 | GCTGCCTCCCGTAGGAGT | Many but not all Bacteria | 5`6-FAM | [37] |
| EUB338-2 | GCAGCCACCCGTAGGTGT | Planctomycetales | 5`6-FAM | [37] |
| EUB338-3 | GCTGCCACCCGTAGGTGT | Verrucomicrobiales | 5`6-FAM | [37] |
| Tet1-266 | CCCGTCGTCGCCTGTAGC | Clone ASM31 | 5`Cy5 | [38] |
| Tet2-892 | TAGTTAGCCTTGCGGCCG | Clone ASM47 | 5`Cy5 | [38] |
| Tet2-174 | GCTCCGTCTCGTATCCGG | *T. jenkinsii*, *T. australiensis*, *T. veronensis*, and *Candidatus* N. limicola | 5`Cy5 | [38] |
| Tet3-654 | GGTCTCCCCTACCATACT | Unclutured *Tetrasphaera* | 5`Cy5 | [38] |

**Supplementary Notes**

**Note S1 Organic substrate transport of *Dermatophilaceae* PAOs**

*Dermatophilaceae* PAOs demonstrated metabolic versatility in other substrate transport mechanisms. Specifically, *Ca.* D. ammonifactor expressed putrescine importer (*puuP*), glycine betaine transporter (*opuD*) and proline-proton symporter (gene *proP*), while *Ca.* L. EBPR_bin.18 expressed maltose/maltodextrin transporter (*malY*) and fucose-proton symporter (*fucP*), and *Ca.* P. EBPR_bin.20 expressed *puup*. Similarly, EBPR_bin.31, a member of the classic *Tetrasphaera_A* genus (clade 2), encoded and expressed *puuP*, *malY*, *fucP*, *opuD*, and L-asparagine permease (*ansP*) (Fig. 3, Fig. 5 and Fig. S6). This diversity in transporter expression suggests potential niche differentiation among PAOs, allowing them to coexist by utilizing distinct carbon sources and metabolic strategies. Such differentiation likely minimizes direct competition while promoting functional complementarity within the EBPR microbial community.

**References:**

1. Wang H, Lin L, Zhang L *et al.* Microbiome assembly mechanism and functional potential in enhanced biological phosphorus removal system enriched with tetrasphaera-related polyphosphate accumulating organisms. *Environ Res*. 2023;**233**:116494 https://doi.org/10.1016/j.envres.2023.116494

2. Wang H, Wang Y, Zhang G *et al.* Temporal dynamics and performance association of the tetrasphaera-enriched microbiome for enhanced biological phosphorus removal. *Engineering*. 2023 https://doi.org/10.1016/j.eng.2022.10.016

3. Andrews SO. Fastqc: A quality control tool for high throughput sequence data.

4. Ewels P, Magnusson M, Lundin S *et al.* Multiqc: Summarize analysis results for multiple tools and samples in a single report. *Bioinformatics*. 2016;**32**:3047-8 https://doi.org/10.1093/bioinformatics/btw354

5. Chen S, Zhou Y, Chen Y *et al.* Fastp: An ultra-fast all-in-one fastq preprocessor. *Bioinformatics*. 2018;**34**:i884-i90 https://doi.org/10.1093/bioinformatics/bty560

6. Schmieder R, Edwards R. Quality control and preprocessing of metagenomic datasets. *Bioinformatics*. 2011;**27**:863-4 https://doi.org/10.1093/bioinformatics/btr026

7. Nurk S, Meleshko D, Korobeynikov A *et al.* Metaspades: A new versatile metagenomic assembler. *Genome Res*. 2017;**27**:824-34 https://doi.org/10.1101/gr.213959.116

8. Kang DD, Li F, Kirton E *et al.* Metabat 2: An adaptive binning algorithm for robust and efficient genome reconstruction from metagenome assemblies. *PeerJ*. 2019;**7**:e7359 https://doi.org/10.7717/peerj.7359

9. Wu Y-W, Tang Y-H, Tringe SG *et al.* Maxbin: An automated binning method to recover individual genomes from metagenomes using an expectation-maximization algorithm. *Microbiome*. 2014;**2**:26 https://doi.org/10.1186/2049-2618-2-26

10. Alneberg J, Bjarnason BS, de Bruijn I *et al.* Binning metagenomic contigs by coverage and composition. *Nature Methods*. 2014;**11**:1144-46 https://doi.org/10.1038/nmeth.3103

11. Uritskiy GV, DiRuggiero J, Taylor J. Metawrap—a flexible pipeline for genome-resolved metagenomic data analysis. *Microbiome*. 2018;**6**:158 https://doi.org/10.1186/s40168-018-0541-1

12. Li D, Luo R, Liu CM *et al.* Megahit v1.0: A fast and scalable metagenome assembler driven by advanced methodologies and community practices. *Methods*. 2016;**102**:3-11 https://doi.org/10.1016/j.ymeth.2016.02.020

13. Olm MR, Brown CT, Brooks B *et al.* Drep: A tool for fast and accurate genomic comparisons that enables improved genome recovery from metagenomes through de-replication. *The ISME Journal*. 2017;**11**:2864-68 https://doi.org/10.1038/ismej.2017.126

14. Chaumeil PA, Mussig AJ, Hugenholtz P *et al.* Gtdb-tk: A toolkit to classify genomes with the genome taxonomy database. *Bioinformatics*. 2019 https://doi.org/10.1093/bioinformatics/btz848

15. Hyatt D, Chen GL, Locascio PF *et al.* Prodigal: Prokaryotic gene recognition and translation initiation site identification. *BMC Bioinformatics*. 2010;**11**:119 https://doi.org/10.1186/1471-2105-11-119

16. Eddy SR. Accelerated profile hmm searches. *PLoS Comput Biol*. 2011;**7**:e1002195 https://doi.org/10.1371/journal.pcbi.1002195

17. Matsen FA, Kodner RB, Armbrust EV. Pplacer: Linear time maximum-likelihood and bayesian phylogenetic placement of sequences onto a fixed reference tree. *BMC Bioinformatics*. 2010;**11**:538 https://doi.org/10.1186/1471-2105-11-538

18. Jain C, Rodriguez-R LM, Phillippy AM *et al.* High throughput ani analysis of 90k prokaryotic genomes reveals clear species boundaries. *Nature Communications*. 2018;**9**:5114 https://doi.org/10.1038/s41467-018-07641-9

19. Price MN, Dehal Ps Fau - Arkin AP, Arkin AP. Fasttree: Computing large minimum evolution trees with profiles instead of a distance matrix.

20. Ondov BD, Treangen TJ, Melsted P *et al.* Mash: Fast genome and metagenome distance estimation using minhash. *Genome Biology*. 2016;**17**:132 https://doi.org/10.1186/s13059-016-0997-x

21. Parks DH, Imelfort M, Skennerton CT *et al.* Checkm: Assessing the quality of microbial genomes recovered from isolates, single cells, and metagenomes. *Genome Res*. 2015;**25**:1043-55

22. Pritchard L, Glover RH, Humphris S *et al.* Genomics and taxonomy in diagnostics for food security: Soft-rotting enterobacterial plant pathogens. *Analytical Methods*. 2016;**8**:12-24 https://doi.org/10.1039/C5AY02550H

23. Team RC. R: A language and environment for statistical computing. 2013

24. Seemann T. Prokka: Rapid prokaryotic genome annotation. *Bioinformatics*. 2014;**30**:2068-9 https://doi.org/10.1093/bioinformatics/btu153

25. Aramaki T, Blanc-Mathieu R, Endo H *et al.* Kofamkoala: Kegg ortholog assignment based on profile hmm and adaptive score threshold. *Bioinformatics*. 2019;**36**:2251-52 https://doi.org/10.1093/bioinformatics/btz859

26. Kanehisa M, Sato Y, Kawashima M *et al.* Kegg as a reference resource for gene and protein annotation. *Nucleic Acids Res*. 2016;**44**:D457-62 https://doi.org/10.1093/nar/gkv1070

27. Buchfink B, Xie C, Huson DH. Fast and sensitive protein alignment using diamond. *Nat Methods*. 2015;**12**:59-60 https://doi.org/10.1038/nmeth.3176

28. Kanehisa M, Sato Y. Kegg mapper for inferring cellular functions from protein sequences. *Protein Sci*. 2020;**29**:28-35 https://doi.org/10.1002/pro.3711

29. Martin M. Cutadapt removes adapter sequences from high-throughput sequencing reads. *2011*. 2011;**17**:3 https://doi.org/10.14806/ej.17.1.200

30. Kopylova E, Noé L, Touzet H. Sortmerna: Fast and accurate filtering of ribosomal rnas in metatranscriptomic data. *Bioinformatics*. 2012;**28**:3211-17 https://doi.org/10.1093/bioinformatics/bts611

31. Kim D, Paggi JM, Park C *et al.* Graph-based genome alignment and genotyping with hisat2 and hisat-genotype. *Nature Biotechnology*. 2019;**37**:907-15 https://doi.org/10.1038/s41587-019-0201-4

32. Putri GH, Anders S, Pyl PT *et al.* Analysing high-throughput sequencing data in python with htseq 2.0. *Bioinformatics*. 2022;**38**:2943-45 https://doi.org/10.1093/bioinformatics/btac166

33. Pertea M, Pertea GM, Antonescu CM *et al.* Stringtie enables improved reconstruction of a transcriptome from rna-seq reads. *Nat Biotechnol*. 2015;**33**:290-5 https://doi.org/10.1038/nbt.3122

34. Mirdita M, Schütze K, Moriwaki Y *et al.* Colabfold: Making protein folding accessible to all. *Nature Methods*. 2022;**19**:679-82 https://doi.org/10.1038/s41592-022-01488-1

35. Liu Y, Yang X, Gan J *et al.* Cb-dock2: Improved protein–ligand blind docking by integrating cavity detection, docking and homologous template fitting. *Nucleic Acids Research*. 2022;**50**:W159-W64 https://doi.org/10.1093/nar/gkac394

36. Ashkenazy H, Abadi S, Martz E *et al.* Consurf 2016: An improved methodology to estimate and visualize evolutionary conservation in macromolecules. *Nucleic Acids Research*. 2016;**44**:W344-W50 https://doi.org/10.1093/nar/gkw408

37. Welles L, Lopez-Vazquez CM, Hooijmans CM *et al.* Prevalence of 'candidatus accumulibacter phosphatis' type ii under phosphate limiting conditions. *AMB Express*. 2016;**6**:44 https://doi.org/10.1186/s13568-016-0214-z

38. Nguyen HT, Le VQ, Hansen AA *et al.* High diversity and abundance of putative polyphosphate-accumulating tetrasphaera-related bacteria in activated sludge systems. *FEMS Microbiol Ecol*. 2011;**76**:256-67 https://doi.org/10.1111/j.1574-6941.2011.01049.x
